# „Essener Transitionsmodell“ bei neuromuskulären Erkrankungen

**DOI:** 10.1007/s00115-022-01274-6

**Published:** 2022-03-07

**Authors:** Michael Fleischer, Bayram Coskun, Benjamin Stolte, Adela Della-Marina, Heike Kölbel, Hildegard Lax, Michael Nonnemacher, Christoph Kleinschnitz, Ulrike Schara-Schmidt, Tim Hagenacker

**Affiliations:** 1 Klinik für Neurologie und Center for Translational Neuro- and Behavioral Science, Universitätsmedizin Essen, Hufelandstraße 55, 45147 Essen, Deutschland; 2 Klinik für Kinderheilkunde 1, Abteilung für Neuropädiatrie, Universitätsmedizin Essen, Hufelandstraße 55, 45147 Essen, Deutschland; 3 Institut für Medizinische Informatik, Biometrie und Epidemiologie, Hufelandstraße 55, 45122 Essen, Deutschland

**Keywords:** Neuromuskuläre Erkrankungen, Late onset Morbus Pompe, Juvenile Myasthenia gravis, Duchenne-Muskeldystrophie, Interdisziplinarität, Neuromuscular Disorders, Late onset Pompe´s Disease, Juvenile Myasthenia Gravis, Duchenne Dystrophy, Interdisciplinarity

## Abstract

**Hintergrund:**

Durch die Optimierung medizinischer Versorgungsstrukturen und die gravierenden Fortschritte bei der Entwicklung neuer Therapieverfahren wird ein Anstieg der Lebenserwartung bei Patienten mit neuromuskulären Erkrankungen beobachtet. Dies führt zu einer Erweiterung des phänotypischen Spektrums, wodurch neue bzw. bislang wenig relevante Krankheitsmanifestationen in unterschiedlichen Organsystemen an Bedeutung gewinnen. Die Betreuung jugendlicher und junger Erwachsener mit neuromuskulären Erkrankungen verlangt daher eine zunehmend enge interdisziplinäre Zusammenarbeit.

**Fragestellung:**

Wie kann der Transitionsprozess von der Pädiatrie in die Erwachsenenmedizin so strukturiert werden, dass die einzelnen Fachdisziplinen effizient in den komplexen Behandlungs- und Versorgungsprozess eingebunden und die Lebensqualität der Patienten verbessert werden?

**Material und Methode:**

An der Universitätsmedizin Essen wurde ein strukturierter Transitionsprozess etabliert. Exemplarisch wurde anhand des Morbus Pompe („late onset M. Pompe“ [LOPD]), der Duchenne-Muskeldystrophie (DMD) und der juvenilen Myasthenia gravis (jMG) ein entsprechendes Versorgungskonzept entwickelt. Dies umfasst vier Elemente: 1) Mit der Einführung klinikübergreifender SOPs („standard operating procedure“) werden die logistischen Abläufe sowie die diagnostischen und therapeutischen Maßnahmen einheitlich abgestimmt und der Transitionsprozess verbindlich festgelegt. 2) Um einen nahtlosen Übergang zu gewährleisten, werden junge Patienten vor Erreichen des 17. Geburtstages mit ihren Eltern im Zuge gemeinsamer Transitionssprechstunden betreut. Dies schafft die Möglichkeit des gegenseitigen Kennenlernens und der Bildung eines nachhaltigen Vertrauensverhältnisses. 3) Ein quartalsweise stattfindendes „Transitionsboard“ bringt die beteiligten Fachdisziplinen aus Kinder- und Erwachsenenmedizin für einen fallbezogenen interdisziplinären Austausch und eine stetige Optimierung des Transitionsprozesses regelmäßig zusammen. 4) Als gemeinsame Informationsplattform und Datengrundlage wurde eine klinikübergreifende „Transitionsdatenbank“, in der medizinische Befunde und Verlaufsparameter erfasst werden, implementiert.

**Schlussfolgerung:**

Mit dem Essener Transitionsmodell soll die Versorgungslücke junger Patienten mit neuromuskulären Erkrankungen während der kritischen Übergangsphase von der Kinder- zur Erwachsenenmedizin geschlossen und die Grundlage für eine erfolgreiche Weiterbehandlung im Erwachsenenalter geschaffen werden.

## Abstract (en)

**Background:**

Advances in healthcare systems with new therapeutic options improve the life expectancy of patients with neuromuscular diseases. With this, a shift in the phenotype of the diseases from the neuromuscular system towards other organs is more frequently observed, requiring closer interdisciplinary cooperation in caring for these young adults. Therefore, the transition to the adult caring system is nowadays a multilayered transfer with the need for complex care of these patients.

**Objective:**

How can the transitional process be efficiently structured to combine the therapeutic effort of each specialist discipline involved and improve the healthcare process and quality of life in young adults with neuromuscular diseases?

**Material and method:**

The Departments of Neuropediatrics and Neurology of the University Medicine Essen established the Essen transition model for a structured transitional process. A concept of care was developed for the late onset Pompe’s disease, Duchenne muscular dystrophy and juvenile myasthenia gravis representatively for neuromuscular diseases. It consists of four components: 1) In a standardized operational procedure (SOP), general processes, clinical diagnostic steps and guidance of treatment between the two departments are harmonized and specified. 2) The young adults and their relatives are seen in a joint consultation of both disciplines allowing a comprehensive handover for healthcare professionals. 3) In a quarterly meeting, transition conference representatives from the different specialized disciplines from pediatric and adult medicine get together for a case-related interdisciplinary exchange. 4) An interdepartmental transitional database was created to integrate all diagnostic results and parameters as a common information platform and data basis.

**Conclusion:**

The Essen transition model aims to close a gap in the transition of patients with neuromuscular diseases and improve healthcare in these patients with their complex needs.

## Hintergrund

Fast 40 % der Kinder und Jugendlichen in Deutschland leiden an einer chronischen Erkrankung, wobei etwa 14 % einen besonderen gesundheitlichen Versorgungsbedarf aufweisen [1]. Durch eine stetige Verbesserung der therapeutischen Möglichkeiten und den Ausbau der Versorgungsstrukturen im Bereich der Neuropädiatrie erreichen immer mehr Jugendliche mit schweren neuromuskulären Erkrankungen das Erwachsenalter [2]. Neuromuskuläre Erkrankungen sind zunehmend als Multisystemerkrankungen zu verstehen. Durch die steigende Lebenserwartung wird eine Erweiterung der phänotypischen Krankheitsspektren mit neuen bzw. bislang im Hintergrund stehenden Organmanifestationen beobachtet [3]. Dies macht eine vertiefte interdisziplinäre und über den Zeitraum der pädiatrischen Betreuung hinausgehende Behandlung notwendig. Um einen möglichst reibungslosen Übergang vom Jugend- in das Erwachsenenalter zu ermöglichen, sollte neben der „allgemeinen“ psychosozialen und persönlichen Entwicklung insbesondere auch die oftmals komplexe krankheitsspezifische medizinische Situation berücksichtigt werden [4]. Da die Patienten inklusive der Eltern bis zum Erreichen des Erwachsenenalters häufig über viele Jahre hinweg – teilweise von ihrer Geburt an – in festen Versorgungsstrukturen betreut wurden, ist ein geordneter Übergang in das System der Erwachsenenmedizin mit möglichst geringem Informationsverlust eine besondere Herausforderung für das Gesundheitssystem und die heranwachsenden Patienten.

Der Begriff „Transition“ beschreibt die Überführung junger Menschen mit chronischen Krankheiten aus der Kinder- und Jugendmedizin in die Erwachsenenmedizin. Der Notwendigkeit eines strukturierten Transitionsprozesses wird gesundheitspolitisch sowohl national als auch international zunehmend Bedeutung beigemessen [1, 5–7]. In Deutschland wurde mit der Gründung einer Gesellschaft für Transitionsmedizin auch eine fachübergreifende Leitlinie erstellt [8]. Erste systematische Programm wie beispielsweise das Berliner Transitionsprogramm existieren bereits, von einer flächendeckenden Versorgung ist man aber noch weit entfernt [9].

Eine unzureichende Transition kann mitunter weitreichende gesundheitliche Folgen für die betroffenen Jugendlichen haben. So kann es ohne eine durchgängige und qualitativ hochwertige interdisziplinäre Betreuung während des Übergangs in die Erwachsenenmedizin neben einer allgemeinen Verschlechterung des Gesundheitszustandes zu schwerwiegenden Komplikationen wie z. B. einer Transplantatabstoßung bei organtransplantierten Patienten kommen [10]. Als weiteres Beispiel mag der Transfer jugendlicher Patienten mit einem Diabetes mellitus Typ 1 dienen; hier verlieren 40 % der Patienten den Kontakt zur qualifizierten Diabetologie, was nachweislich in einem ansteigenden Risiko für Hyperglykämien resultiert [11]. Verglichen mit Jugendlichen, die noch in der pädiatrischen Versorgung verblieben waren, wiesen Jugendliche nach dem Transfer in die Erwachsenenmedizin ein 2,5-fach erhöhtes Risiko für eine ungünstige Blutzuckereinstellung mitsamt entsprechenden Folgeschäden auf. Umgekehrt konnten strukturierte Transitionsprogramme mit individuellem Fallmanagement die Rate von Folgeschäden von 40 % auf etwa 10 % reduzieren [12].

Im klinischen Alltag verläuft die Transition häufig als ungeplanter Prozess, sobald eine Weiterbehandlung im pädiatrischen Bereich nicht mehr möglich ist. Dies geschieht zumeist an einem „Stichtag“, in der Regel mit Erreichen der Volljährigkeit, oder im ungünstigsten Fall gar im Zuge eines medizinischen Notfalles [13]. Dies führt oftmals zu einer unstrukturierten und unvollständigen Übergabe und wird der komplexen medizinischen und sozialen Situation der Betroffenen nicht gerecht. Hierdurch wird auch der Erfolg vorausgegangener langjähriger therapeutischer Bemühungen gefährdet, was mitunter weitreichende medizinische und soziale Folgen nach sich ziehen kann [14, 15]. Etwa 30–40 % der betroffenen Jugendlichen bestätigen Probleme hinsichtlich der Transition [1]. Eine misslungene Transition kann allerdings nicht nur für den Einzelnen negative gesundheitliche Folgen haben, sondern ist auch gesundheitspolitisch und gesellschaftlich von hoher Relevanz. Durch eine gezielte Begleitung in die Erwachsenenmedizin können das Risiko für Komplikationen, Hospitalisierung, Folgeerkrankungen, Frühberentung und dadurch letztlich auch die Kosten für das Gesundheits- und Rentensystem reduziert werden [16]. Strukturierte Transitionsprogramme sind daher als Chance zu verstehen, eine verbesserte und auch ressourcenschonende Behandlung sicherzustellen [17].

Zusammenfassend ist eine gelungene Transition durch eine strukturierte und koordinierte Überführung jugendlicher Patienten in die Erwachsenenmedizin gekennzeichnet. Dies erfordert einen Aufbau kontinuierlicher, interdisziplinärer und patientenorientierter Versorgungsstrukturen hoher Qualität. Derzeit findet eine solche strukturierte Transition allerdings nur selten statt. Für die Transition von Patienten mit neuromuskulären Erkrankungen wurde das „Essener Transitionsmodell“ etabliert, das im Folgenden dargestellt wird.

## Herausforderungen der Transition

Die wesentliche Herausforderung einer erfolgreichen Transition besteht darin, zahlreiche einzelne Elemente zu koordinieren und so ineinandergreifen zu lassen, dass ein effizienter, im klinischen Alltag machbarer und dennoch qualitativ hochwertiger Gesamtprozess gelingt. In einem Gutachten des Sachverständigenrates zur Begutachtung der Entwicklung im Gesundheitswesen aus dem Jahre 2009 wurden zwei wesentliche Problemfelder der Transition identifiziert. Zum einen wurden Schwierigkeiten auf Systemebene in Form von Finanzierungs- und Organisationsdefiziten und zum anderen Defizite auf fachlicher und sozialer Ebene dargestellt.

Ein wichtiger Systemfaktor, der die Transition behindert, ist die nicht ausreichende Vergütung des erhöhten Behandlungsaufwandes von Adoleszenten in besonderen Betreuungssituationen. So werden Patienten in der Zeit der Transition von mindestens zwei Fachärzten der jeweiligen Disziplin zeitgleich behandelt. Weitere systemische Hindernisse bestehen in einem Mangel an qualifiziertem Personal, fehlenden Schulungen für Betreuer und dem Fehlen einheitlicher Regelungen zum optimalen Zeitpunkt der Einleitung des Transitionsprozesses (Sachverständigenrat zur Begutachtung der Entwicklung im Gesundheitswesen 2009). Angesichts dieser Tatsachen ist insbesondere eine flächendeckende, spezifische Aus- und Weiterbildung des medizinischen Personals von hoher Bedeutung [18–20]. Die Weiterbildung auf dem Gebiet der Transition für Fachärzte ist in anderen Ländern, wenngleich bislang auch nur in Ansätzen, bereits geregelt. So wurde beispielsweise in Großbritannien ein Kurrikulum (Adolescent Health Programme) entwickelt, das als Onlineprogramm für Fachärzte zugänglich ist [21].

Auf fachlicher Ebene fühlen sich die Behandler aus der Erwachsenenmedizin auf die Betreuung chronisch erkrankter Jugendlicher oftmals nicht adäquat vorbereitet [22]. Im Vergleich zur Kinder- und Jugendmedizin ist die Behandlung Erwachsener oftmals in einem höheren Maße krankheitsorientiert und weniger „ganzheitlich“ personenbezogen bzw. individuell [23]. Das kann für den Patienten eine ungewohnte Situation darstellen, die zu Unsicherheiten führen kann. Ein weiterer wichtiger Aspekt sind die Eltern der Patienten, die durch ihre, wenngleich wohlgemeinte, Fürsorge die persönliche Weiterentwicklung und Selbstständigkeit junger Erwachsener auch im medizinischen Bereich beeinträchtigen können [24, 25]. Auch kann die Beziehung zwischen Erziehungsberechtigten und bisherigen Behandlern insofern ein Hindernis darstellen, als die „Trennung“ im Falle eines besonders vertrauten Verhältnisses auch auf dieser Ebene schwerfallen kann [26]. Dies ist eine neue Herausforderung für die Behandler aus der Erwachsenenmedizin, die im Rahmen der Transition mit drei (Patient und Eltern) Ansprechpartnern konfrontiert werden.

## Das Essener Modell – für eine erfolgreiche Transition

**Figure 1:**
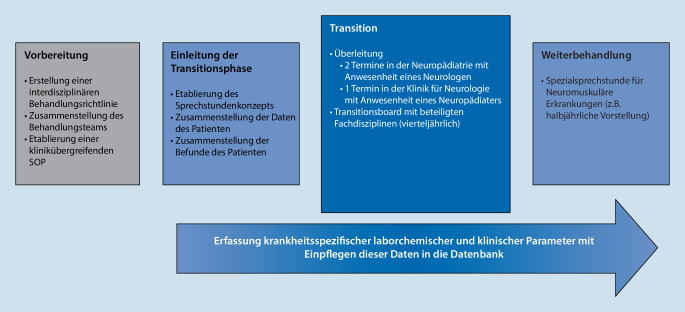

In der Universitätsmedizin Essen wurde ein strukturierter Transitionsprozess für Patienten mit neuromuskulären Erkrankungen etabliert, das am Beispiel des Morbus Pompe („late onset M. Pompe“ [LOPD]), der Duchenne-Muskeldystrophie (DMD) und der juvenilen Myasthenia gravis (jMG) evaluiert wurde (Abb. 1).

Das „Essener Modell“ setzt sich aus folgenden Elementen zusammen:

1. klinikübergreifende SOPs („standard operating procedure“),
2. Transitionssprechstunde,
3. interdisziplinäre Fallkonferenz („Transitionsboard“),
4. Transitionsdatenbank.

### Klinikübergreifende SOPs

Durch die direkt am Behandlungsprozess beteiligten Behandler aus den jeweiligen Fachabteilungen wurden klinikübergreifende SOPs erstellt. Hierdurch wurden die Durchführung diagnostischer Schritte sowie die Indikationsstellung und Durchführung spezifischer Therapien standardisiert. Die SOPs sollen beispielsweise sicherstellen, dass bei Patienten mit gesicherter Diagnose eines LOPD unter Durchführung einer Enzymersatztherapie (EET) klinikübergreifend standardisierte Untersuchungen mit einheitlicher Erfassung der Befunde erfolgen. Der Prozess der SOP-Erstellung wurde vom klinikeigenen Qualitätsmanagement begleitet, das die SOPs allen Beteiligten in einem Dokumentmanagementsystem zur Verfügung stellt.

### Transitionssprechstunde

Die Identifikation und Aufnahme für bzw. in den Transitionsprozess erfolgen mit Vollendung des 16. Lebensjahres. In einer Transitionssprechstunde werden die Jugendlichen und deren Angehörigen durch ein Behandlungsteam, bestehend aus jeweils einem ärztlichen Vertreter der Neuropädiatrie und Neurologie, untersucht und der bisherige Verlauf und die vorangegangene Behandlung besprochen und das weitere diagnostische und therapeutische Vorgehen besprochen. Durch zwei gemeinsame Untersuchungstermine innerhalb von 12 Monaten ist die Möglichkeit gegeben, dass die Patienten und Angehörigen den Weiterbehandler als künftigen Interaktionspartner kennenlernen können und mögliche Schwierigkeiten beim Übergang von der Kinder- und Jugendmedizin in die Erwachsenenneurologie rechtzeitig identifiziert werden können. Im Rahmen der Transitionssprechstunde werden neben den für die Fortführung der Diagnostik und Therapie notwendigen Schritte auch die zukünftig etwaig notwendige Mitbetreuung durch andere Fachdisziplinen geplant und koordiniert (z. B. Pneumologie, Kardiologie).

Mit Erreichen des 18. Geburtstages wird dann die weitere Betreuung durch die neuromuskuläre Ambulanz im Erwachsenenbereich übernommen und die Patienten gemäß den dortigen Behandlungsstandards weiter versorgt.

### Interdisziplinäre Fallkonferenz (Transitionsboard)

In anderen Fächern wie der Onkologie sind interdisziplinäre Fallkonferenzen (Tumorboards) als Instrument für die Steuerung der individuellen Behandlung bereits fest etabliert. Analog dazu kann eine Fallkonferenz im Bereich der neuromuskulären Erkrankungen eine Koordination interdisziplinärer diagnostischer und therapeutischer Maßnahmen ermöglichen. Dementsprechend wurde ein Transitionsboard etabliert, das quartalsweise stattfindet und die jeweiligen Fachdisziplinen aus dem pädiatrischen und Erwachsenenbereich umfasst. Dies unterstützt einen effizienten Arbeitsablauf innerhalb der Transition. Die Ergebnisse des Transitionsboards werden fallbezogen dokumentiert und in der Transitionsdatenbank hinterlegt.

### Transitionsdatenbank

Grundlage einer erfolgreichen Transition ist der effektive Informationsaustausch über einen oftmals langen Krankheitsverlauf, und zwar über verschiedene Behandlungsdisziplinen hinweg. Hierzu bedarf es einer gemeinsamen Plattform, die klinikübergreifend allen Therapeuten Behandlungs- und Verlaufsparameter zur Verfügung stellt. Neben den Basisdaten der Patienten werden Diagnose- und Funktionsparameter im Verlauf, weitere therapierelevante Informationen, Angaben zur Hilfs- und Heilmittelversorgung sowie sozialmedizinische Informationen bereitgestellt. Um einen krankheitsbildspezifischen Schwerpunkt zu erhalten und den Aufbau einer Parallelstruktur zum bereits bestehenden Krankenhausinformationssystem zu vermeiden, wurden die notwendigen Parameter und Daten im Rahmen einer Konsensbildung mit den beteiligten Fachdisziplinen abgestimmt.

Die für neuromuskuläre Erkrankungen spezifisch erhobenen Parameter umfassen beispielsweise Körpergröße, Gewicht, BMI, Bauchumfang, Laborparameter (CK, CK-MB, Leberenzyme, Nierenwerte, Antikörpertiter gegen Alfaglucosidase bei LOPD) und humangenetische Befunde. Zur Beurteilung des Verlaufs muskuloskeletaler Symptome werden neben Einzelkraftgraden die Umfänge der oberen und unteren Extremitäten und motorische Bewertungs- und Verlaufsscores (z. B. motorische Meilensteine, Brooke Upper Extremity Scale für die Funktion der oberen Extremitäten, 6‑minute-walking-Test zur Beurteilung der Gehfähigkeit und muskulären Ausdauer) erfasst. Die pulmonale und kardiale Leistungsfähigkeit wird über die Messung der Vitalkapazität (VC), forcierten Einsekundenkapazität (FEV1), Ejektionsfraktion (EF) und fraktionellen Verkürzung (FS) abgebildet.

Durch ein Vorstellungsintervall von 6 Monaten ergibt sich ein umfassendes Zustandsbild, welches eine Beurteilung des Krankheitsverlaufes und oftmals auch das Ansprechen auf die Therapie im Längsschnitt erlaubt. Umgekehrt wird die Notwendigkeit von Interventionen oder weiteren therapeutischen Maßnahmen frühzeitig erkannt. Die Aufnahme von Information über die Hilfs- und Heilmittelversorgung sowie die Erfassung sozialmedizinischer Aspekte sollen das Gesamtbild über den individuellen Patienten abrunden und einer Unter- oder Überversorgung vorbeugen.

## Ausblick: wissenschaftliche Betrachtung des Transitionsprozesses

**Figure 2:**
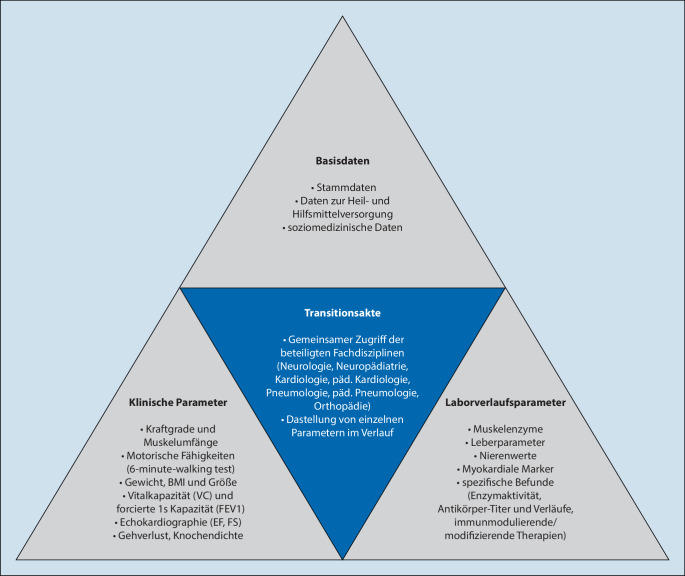

**Figure 3:**
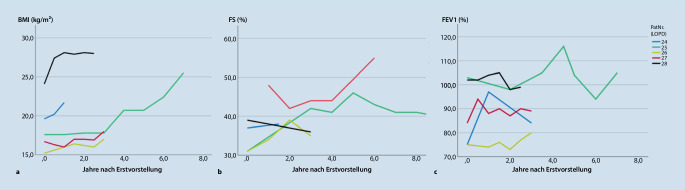

Die Erstellung der Transitionsdatenbank schafft eine Informationsplattform für alle beteiligten Behandlungspartner innerhalb der Universitätsmedizin, indem sie die Erfassung von Kenngrößen und Verlaufsparametern ermöglicht (Abb. 2). Mittels einer visuell übersichtlich gehaltenen Maske und Voreintragungen wird der Aufwand bei der Dokumentation gering gehalten. In der Pilotphase wurden die vorhandenen Daten für die ausgewählten Modellkrankheitsbilder (LOPD, DMD und jMG) retrospektiv erfasst. Neben demographischen Informationen (Tab. 1) ist eine exemplarische Abfrage einiger klinischer Verlaufsparameter (Lungenfunktion, fraktionelle Verkürzung und BMI; Abb. 3) dargestellt. Neben den Verlaufsparametern ist die Erarbeitung eines Transitionsscores geplant. Darin sollen die pädiatrischen Verlaufsparameter und Bewertungsscores in die Erwachsenenmedizin übersetzt werden. Damit könnte ein Parameter für die Bewertung der Transitionsqualität etabliert werden. Krankheitsverläufe ließen sich über die Zeit besser dokumentieren und die Notwendigkeit z. B. einer Therapieumstellung während der Adoleszenz leichter erkennen.

**Table 1:** 

	LOPD	DMD	jMG
*Anzahl*	10	20	5
*Geschlecht*			
Männlich	6	20	–
Weiblich	4	–	5
*Alter*	30,1	24,9	22,2
*BMI*	22,0	23,6	21,5

*BMI* Body-Mass-Index

## Fazit für die Praxis

- Durch eine bessere Versorgung und neue Therapieoptionen verlängert sich die Lebenserwartung bei Patienten mit neuromuskulären Erkrankungen und es bilden sich neue Phänotypen aus, die bislang noch nicht beobachtet werden konnten.
- Ein enger Austausch zwischen den behandelnden Fachdisziplinen ist aufgrund der komplexen neuromuskulären Krankheitsbilder wichtig.
- Ein strukturierter Transitionsprozess verbessert die Behandlungsadhärenz und die Lebensqualität der jungen Patienten.
- Das „Essener Transitionsmodell“ umfasst einzelne Komponenten, die als Vorlage für andere Zentren zur Etablierung eines strukturierten Transitionsprozesses mit Anpassung an die lokalen infrastrukturellen Gegebenheiten dienen können.
- Für die Zukunft sind auch Empfehlungen aus der S3-Leitlinie zur Transition sinnvoll in den Prozess einzubeziehen.
